# Carbon Balance and Contribution of Harvested Wood Products in China Based on the Production Approach of the Intergovernmental Panel on Climate Change

**DOI:** 10.3390/ijerph13111132

**Published:** 2016-11-12

**Authors:** Chunyi Ji, Wenbin Cao, Yong Chen, Hongqiang Yang

**Affiliations:** 1 School of Business, Jiangnan University, 1800 Lihu Avenue, Wuxi 214122, China; jcy_830226@sina.com; 2 Research Institute of Forestry Policy and Information, China Academy of Forestry, Beijing 100091, China; chenyong2000@vip.sina.com; 3 College of Economics and Management, Nanjing Forestry University, Nanjing 210037, China; yhqnfu@gmail.com; 4 Center for the Yangtze River Delta’s Socioeconomic Development, Nanjing University, Nanjing 210093, China; 5 Research Center for Economics and Trade in Forest Products of the State Forestry Administration, Nanjing 210037, China

**Keywords:** IPCC framework, harvested wood products, carbon substitution, carbon balance

## Abstract

The carbon sequestration of harvested wood products (HWP) plays an important role in climate mitigation. Accounting the carbon contribution of national HWP carbon pools has been listed as one of the key topics for negotiation in the United Nations Framework Convention on Climate Change. On the basis of the revised Production Approach of the Intergovernmental Panel on Climate Change (2013) (IPCC), this study assessed the accounting of carbon stock and emissions from the HWP pool in China and then analyzed its balance and contribution to carbon mitigation from 1960 to 2014. Research results showed that the accumulated carbon stock in China’s HWP carbon pool increased from 130 Teragrams Carbon (TgC) in 1960 to 705.6 TgC in 2014. The annual increment in the carbon stock rose from 3.2 TgC in 1960 to 45.2 TgC in 2014. The category of solid wood products accounted for approximately 95% of the annual amount. The reduction in carbon emissions was approximately twelve times that of the emissions from the HWP producing and processing stage during the last decade. Furthermore, the amount of carbon stock and emission reduction increased from 23 TgC in 1960 to 76.1 TgC in 2014. The annual contribution of HWP could compensate for approximately 2.9% of the national carbon dioxide emissions in China.

## 1. Introduction

Under the United Nations Framework Convention on Climate Change, any process, activity, or mechanism that removes carbon from the atmosphere is referred to as a “carbon sink” [1]. The role of harvested wood products (HWP) in mitigating climate change has long been recognized. HWP can contribute through three main routes: using wood products as carbon stock, using biomass for energy, and substituting for energy-intensive materials [2].

Vigorous discussions about HWP in relation to climate change are ongoing and are mainly focused on approaches that account for HWP carbon pools. The Intergovernmental Panel on Climate Change (IPCC) has proposed three approaches for HWP carbon accounting in the 2006 IPCC Guidelines for National Greenhouse Gas Inventories; these approaches are the stock-change approach, the atmospheric flow approach, and the production approach (referred to as PA-2006 hereafter). These approaches differ in terms of their treatment of the HWP international trade, engendering different accounting scope and system boundary of the HWP carbon pool [1]. However, the 2006 Guidelines did not show preference for any of these approaches. The national carbon accounting using any one of these approaches would be acceptable. Under the IPCC Guidelines, studies on estimating the HWP carbon pool have been conducted worldwide [3,4] in numerous districts and individual countries, including Annex 1 Parties [5], Finland [6], USA [7,8], Russia [9], China [10], Ireland [11], and Portugal [12]. The research results verify that HWP plays an important role in climate mitigation. Meanwhile, numerous studies have promoted the evolution of the three IPCC approaches and their applications [11,12,13,14]. A different choice of the approaches could generate different national carbon mitigation results, socioeconomic and environmental implications and impacts. 

The 17th Conference of Party (COP17) held in Durban in 2011 concluded that only HWP from domestic harvests could be included in national greenhouse gas (GHG) inventories. The 2013 Revised Supplementary Methods and Good Practice Guidance Arising from the Kyoto Protocol (referred to as 2013 Guidance hereafter) did not repudiate other approaches, although it selected PA (referred to as PA-2013 hereafter) as the universal approach [15]. Under this guidance, numerous scholars have adopted PA-2013 to investigate carbon stock in the forestry carbon pool and the life cycle of HWP [16]. A few scholars combined PA with carbon flow theory to develop new model architectures, in order to estimate the contribution of both carbon stock and substitution of wood use for energy or energy-intensive material. Chihiro et al. [17] constructed a system dynamic model to investigate the interregional carbon flows and balance of HWP in Japan. When the carbon reduction of wood substitution for energy was considered, the sum of the carbon sink and reduction were greater than the carbon emissions associated with HWP. 

China is both the major supplier and consumer of wood resources in the world. Recent studies on the national HWP carbon sink have mainly focused on estimating the carbon removal achieved by the country’s HWP carbon pool [10] and analyzing the HWP carbon pool structure [18]. Researchers have determined that HWP has become a significant component of the national carbon pool and have proposed that the carbon balance of HWP should be included in the national GHG inventories. However, different selected methods will affect the research results. Furthermore, the current carbon flow and balance of HWP in China have not yet been properly and comprehensively investigated. Therefore, our study aims to assess and analyze the carbon balance and contribution of the HWP carbon pool in China, on the basis of elaborating the mechanisms of carbon flow, including carbon sequestration, carbon emission, and substitution of fossil fuel consumption.

## 2. Basic Mechanism

### 2.1. HWP Carbon Stock

**Figure 1:**
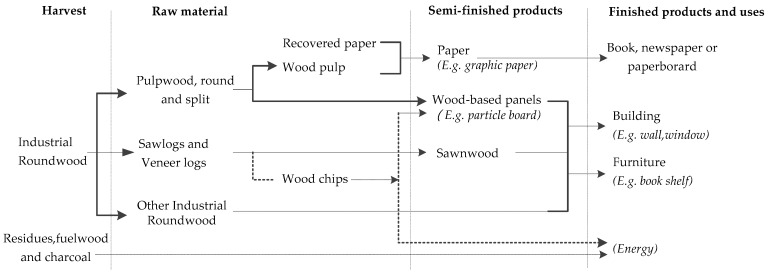
Different processing stages of wood products along the harvested wood products (HWP) process and value chain.

Long-term sustainable forest management and timely transfer of carbon into wood products help reduce atmospheric carbon. Along the HWP process and value chain, the flow of an HWP carbon pool can be divided into three stages (Figure 1). First, in the harvest process, carbon absorbed by forests during photosynthesis will be transferred to carbon storage in industrial roundwood, wood residues, fuelwood, and charcoal, which can be regarded as an input of the HWP carbon pool. Second, in the HWP processing stage, carbon in industrial roundwood will be transferred to carbon sink in different categories of wood products and then released gradually over a long life cycle. Meanwhile, carbon in residues and fuelwood/charcoal will be released through decay or combustion within a short period. Third, in the waste disposal stage, carbon stored in wood waste discarded in landfills will be preserved for a long period under anaerobic conditions. 

The carbon uptake of the HWP in use will be released during the life cycle of different categories of finished wood products, such as paper products, buildings, or furniture. Carbon stock can be calculated by multiplying usage amounts of wooden products with a lifetime function, which is established from various parameters, including number of years passed, decrease rate, and half-lives. HWP can become a considerable carbon stock through effective use and management. 

### 2.2. HWP Carbon Balance

#### 2.2.1. Carbon Substitution

Carbon emissions can be reduced when wood products are used for construction and as substitutes for energy-intensive materials [19]. Meanwhile, wood residues and fuelwood/charcoal can be used as substitutes for fossil fuels, as biomass can be used as renewable energy sources. The carbon in these materials will be released through combustion within a short period. 

#### 2.2.2. Carbon Emission

In the processes of producing raw wood materials, semi-finished products, and end-use products, the consumption of electricity and fuels will increase CO_2_ emissions (Teragrams Carbon (TgC)). Carbon emissions are calculated by multiplying the production amounts (m^3^) of wood products by their respective carbon emission intensities (TgC/m^3^) [17]. These emission intensities are calculated by dividing direct carbon (CO_2_) emissions (TgC) from the energy consumption for each sector by the production amounts (m^3^). Direct carbon emissions are calculated through an input–output (I–O) analysis based on national I–O tables, which divide the economy of a nation into several sectors. 

### 2.3. HWP Carbon Contribution

The accounting of HWP is confined to products where wood is derived from domestic harvest in the reporting country. In most previous studies, the estimation of HWP contribution to carbon mitigation refers to the annual change in the carbon stock of the HWP in use. In this research, we expand the system boundary of HWP carbon flow to assess the carbon stock of the HWP in use, besides the balance between the carbon emission reduction by wood substitution for fossil fuel and carbon emission increase in the production and processing stages. Then, net carbon removal (carbon stock and net emission reduction) can be estimated as a consecutive carbon contribution of China’s HWP to compensate for national carbon emissions. 

## 3. Methodology and Data

### 3.1. Carbon Stock and Its Annual Change

The 2013 Revised Guidance of the IPCC established PA-2013 as the universal approach. It provided good practice guidance for estimating annual changes in carbon stocks and associated carbon emissions and removals from the HWP pool [15]. The equations and mathematical explanation of PA-2013 for estimating the accumulated changes in the carbon pool are presented as follows:

(1) $$\Delta C_{STC}(i)=C_{STC}(i+1)-C_{STC}(i)$$

(2) $$C_{STC}(i+1)=e^{-k}\cdot C_{STC}(i)+[\frac{\left(1-e^{-k}\right)}{k}]\cdot Inflow(i)$$

(3) $$Inflow(i)=P\cdot f_{DP}(i)$$

(4) $$f_{DP}(i)=\left\{ \begin{array}{l} f_{IRW}(i),\;for\;sawnwood\;and\;wood-based\;panels\;\\ f_{IRW}(i)\cdot f_{PULP}(i),\;for\;paper\;and\;paperboard \end{array}\right.$$

(5) $$f_{IRW}(i)=\frac{IRW_{P}(i)-IRW_{EX}(i)}{IRW_{P}(i)+IRW_{IM}(i)-IRW_{EX}(i)}$$

(6) $$f_{PULP}(i)=\frac{PULP_{P}(i)-PULP_{EX}(i)}{PULP_{P}(i)+PULP_{IM}(i)-PULP_{EX}(i)}$$

(7) P=V⋅D⋅R

(8) $$V_{t}=V_{1961}\cdot e^{\left[U\cdot (t-1961)\right]}$$

where ∆*C_STC_*(*i*) is the carbon stock change in the HWP pool in year *i*; *C_STC_*(*i*) is the carbon stock of the HWP pool in year *i*; k is the decay constant of the first-order decay (*k* = *ln*(2) / *HL*, where *HL* is half-life of the HWP pool in years. A half-life is the number of years it takes to lose one-half of the material currently in the pool); *Inflow(i)* is the inflow to the HWP pool during year *i*; *P* is the carbon in annual domestic production of solid wood or paper products in year *i*; *f_DP_*(*i*) is the share of domestic feedstock for the production of a particular HWP category originating from domestic forests in year *i*; *f_IRW_*(*i*) is the share of domestic industrial roundwood feedstock for the production of solid wood products in year *i*; *f_PULP_*(*i*) is the share of domestic pulp feedstock for the production of paper products in year *i*; *IRW_P_(i)*, *IRW_IM_(i)* and *IRW_EX_(i)* are the carbon amounts in industrial roundwood production, imports, and exports, respectively, in year *i*; *PULP_P_(i)*, *PULP_IM_(i)* and *PULP_EX_(i)* are the carbon amounts in industrial roundwood production, imports, and exports, respectively, in year *i*; *V* is the production, imports or exports, in year *i*; *D* is the basic density of HWP; *R* is the carbon fraction; and *U* is the estimated continuous rate of change in industrial roundwood consumption for the region that includes the reporting country between 1900 and 1961 (in Asia, *U* = 0.0217). 

### 3.2. Carbon Substitution for Fossil Fuel

The fossil fuel substitution factor is used to estimate carbon substitution for fossil fuels. The value is equal to the reduction amount of carbon emissions when wood products that contained 1 TgC is used as a substitute for fossil fuels to produce energy.

(9) $$C_{SUB}(i)=P\cdot D_{f}$$

(10) $$D_{f}=\;\frac{C_{f}/E_{f}}{C_{b}/E_{b}}$$

where *C_SUB_*(*i*) is the carbon substitution of the HWP in year *i*; *P* is the carbon in annual domestic production of wood fuel, residue, or wood charcoal in year *i*; *D_f_* is the fossil fuel substitution factor; *C_b_* is the carbon emission from biofuel combustion (TgC/Terajoule (TJ)); *C_f_* is the carbon emission from fossil fuel combustion; *E_b_* is the energy consumption efficiency of biofuel (%); and *E_f_* is the energy consumption efficiency of fossil fuels.

To compare the energy conversion efficiencies of biofuels and fossil fuels, many important issues, such as the energy systems required for the production of energy, quality loss in the production and transport processes, energy and land use for the production of bioenergy and other environmental issues, should be considered [20]. To simplify the study, we assume the same energy conversion efficiency and consider carbon emissions in the production of both biofuels and fossil fuels. The *D_f_* factor is derived as follows:

(11) $$D_{f}=\frac{C_{f}+P_{f}}{C_{b}+P_{f}}$$

where *P_b_* is the carbon emission in the biofuel production process; and *P_f_* is the carbon emission in the fossil fuel production process.

### 3.3. Data Acquisition

The IPCC Guidance suggest that the statistics of different categories of HWP should be obtained from FOASTAT of the Food and Agriculture Organization (FAO). HWPs are divided according to different decay rates into solid wood products (industrial roundwood, sawnwood, and wood-based panels) and paper products (paper and paperboard). The statistics of production, import, and export volumes for various types of HWP are obtained in 1960–2014.

This study adopts the factors provided in the IPCC 2013 Appendix, which are the universal data sources for HWP carbon accounting (see Table A1). The carbon emission intensity and substitution factors for HWP carbon balance system are shown in Table A2. The basic density of HWP is the oven-dry species tons per m^3^ of solid wood product or oven-dry per air dried ton of pulp or paper product. The carbon content of HWP refers to tons carbon per m^3^ of solid wood products or per air-dried ton carbon of paper products. This amount can be calculated by multiplying the density of each material by the carbon conversion factor [15]. 

## 4. Results

### 4.1. Carbon Stock and Annual Change in the HWP Pool

**Figure 2:**
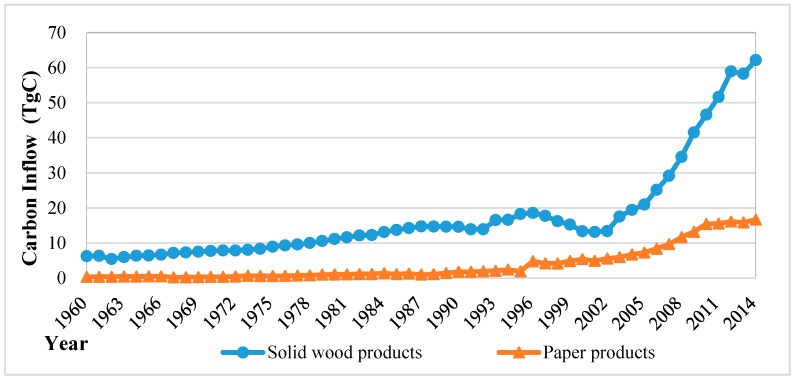
Carbon inflow of two categories of HWP from harvest in China from1960 to 2014.

Without considering decay of wood products, the annual carbon inflow of two categories of wood products to the HWP carbon pool during the period of 1960–2014 is shown in Figure 2. 

In comparison, the total carbon inflow of the paper products was 22.3% of the solid wood products over the past 50 years. The annual carbon inflow of paper products gradually increased from 0.3 million TgC (1.1 Tg CO_2_) in 1960 to a peak of 16.7 TgC in 2014. This amount was projected to continuously increase to 31 TgC in 2030. The amount of the carbon inflow of solid products increased from 6.3 TgC in 1960 to 18.5 TgC in 1997, and then slightly declined to 13.1 TgC in 2001 because of the implementation of national forest protection policies. Then, it increased sharply from 2002 and reached 62.2 TgC in 2014.

**Figure 3:**
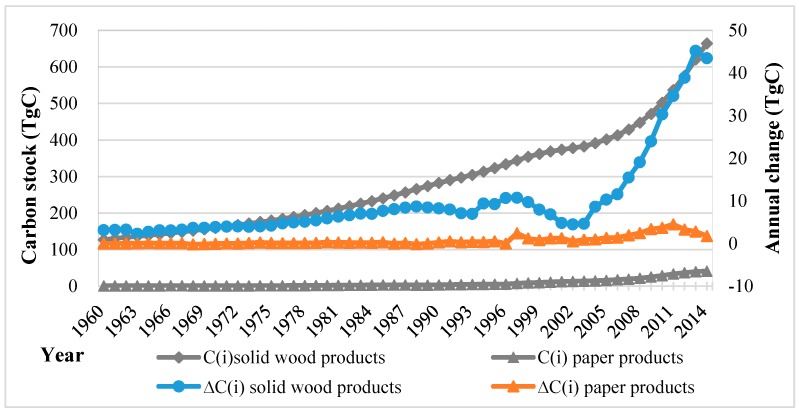
Carbon stock and annual change of two categories of HWP from harvest in China from 1960 to 2014.

The amount of wood material in use will actually decrease over the entire life cycle. The outflow from the HWP carbon pool was calculated based on estimated half-life (solid wood products, 30 years; paper products, 2 years) and associated decay rates of HWP from use by assuming first-order decay rates. We provide valid estimates of the carbon stock and total release of carbon from HWP. The accumulated carbon stock increased gradually from 130 Tg in 1960 to 705.6 Tg in 2014, with the contributions of solid wood products and paper products being 664.2 TgC (94.1%) and 41.4 TgC (5.9%), respectively (Figure 3). The annual change in the carbon stock rose from 3.2 TgC in 1960 to 45.2 TgC in 2014. The category of solid wood products fluctuated before 2003, underwent a sharp increase to 43 TgC in 2014, and reached approximately 95% of the total annual carbon increment of the HWP carbon pool. 

### 4.2. Carbon Balance and Contribution to Climate Mitigation

**Figure 4:**
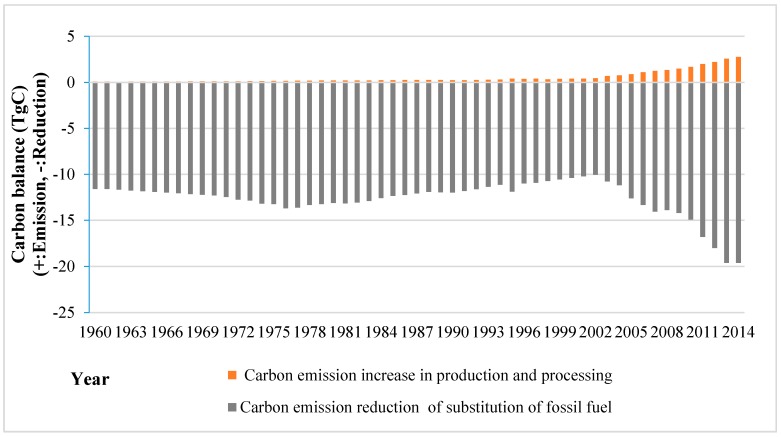
Carbon balance between carbon substitution and carbon emission of China’s HWP from 1960 to 2014.

Figure 4 shows the annual carbon balance of HWP, including carbon balance between emission reduction via substitution of fossil fuel (coal) with wood residue or charcoal, as well as carbon emissions caused by fossil fuel consumption in the production and processing of wood products. Carbon emissions increased from 0.08 TgC in 1960 to 2.8 TgC in 2014. An increase in carbon reduction was observed from 19.9 TgC in 1960 to 33.7 TgC in 2014, which was 1.7 times the 1960 value, by using wood fuel, residue and wood charcoal for energy. 

**Figure 5:**
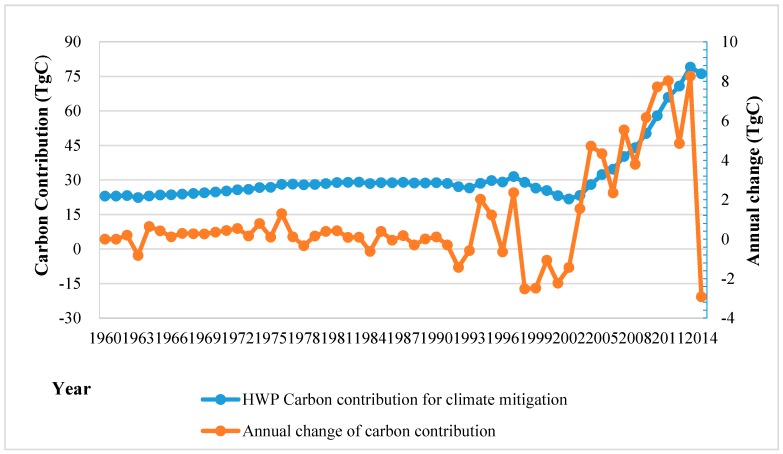
Carbon contribution of HWP for climate mitigation in China from 1960 to 2014.

To compensate for national carbon emissions, the net carbon contribution of China’s HWP was approximated based on the estimation of the value of the carbon stock of HWP; the carbon emission reduction by substitution for fossil fuels; and the value of carbon emissions increase in the production and processing stages. The carbon contribution increased from 23 TgC in 1960 to 76.1 TgC in 2014 (Figure 5). The annual change of the amount showed an obvious 11 TgC reduction in 2014. The international energy statistics published in the U.S. Energy Information Administration show that CO_2_ emissions from the consumption of energy in China increased from 1440 Tg in 1981 to 8687 Tg in 2013. One unit of C sequestered in the HWP pool can be equal to 44/12 unit of CO_2_ removed from the atmosphere. Notably, in the last decade, the annual contribution of HWP could compensate for approximately 2.9% of the national CO_2_ emissions as the annual carbon stock and carbon reduction by substitution of fossil fuels maintained an evident increase. 

## 5. Discussion

### 5.1. Carbon Stock in Wood Products

Previous research reported that the amount of national carbon stock in HWP was 519.9 TgC, with an annual increment of 13.53 TgC in 2011 [10]. Recent research results showed that the carbon stock was 470 TgC with an annual increment of 40 TgC in 2014 [21]. In the current research, the result of national carbon stock in the HWP pool is 570 TgC in 2011 and 706 TgC in 2014, which is higher than those of the existing researches. This result is mainly attributed to the different coefficients, which describe the relationship between HWP trade and production in PA-2006 and the relationship between the corresponding feedstock trade and production in PA-2013. The share of domestic feedstock for the production of wood products originating from domestic forests is used in the PA-2013 methodology. 

China is a major importing country of industrial roundwood, with the carbon stock in imported wood being one-third that of domestic wood. If the feedstock of Chinese domestic industry roundwood is increased or increasingly used as a substitute for net imported wood, then the amount of national HWP carbon stock will significantly increase. 

In addition, the amount of carbon stock in the HWP pool is affected by the updated FAO statistical data and conversion factors. If the life cycle of solid wood products and paper products can be extended by factors, such as policies that promote the recycling of wood, then the national carbon stock of HWP will also increase. 

### 5.2. Carbon Balance and Contribution

The contribution of wood usage to produce energy is considerable. The reductions in carbon emissions were about 12 times the size of the emissions in HWP producing and processing stage during the past decade (Figure 5). If wood is substituted for natural gas instead of coal, the reduction amount of carbon emissions will drop by approximately 40% due to the different energy conversion efficiency values. Nevertheless, the reductions were still much greater than the carbon emissions. The effects of substitution of energy would be further enhanced if the energy conversion efficiency of waste wood and residues can be improved. 

## 6. Conclusions

This study analyzed the mechanisms of carbon sequestration, carbon emissions, and carbon substitution associated with carbon flow in HWP pools. Then, we estimated the accounting and annual change of China’s HWP carbon pool and analyzed its contribution to carbon mitigation from 1960 to 2014.

China’s accumulated carbon stock of solid wood products and paper products in the HWP carbon pool increased from 130 TgC in 1960 to 705.6 TgC in 2014. The annual change in carbon stock of solid wood products increased from 3.2 TgC in 1960 to 43.5 TgC in 2014, reaching approximately 95% of the total annual carbon increment of the HWP carbon pool.

Considering the carbon balance between emission reduction via substitution of fossil fuels and carbon emissions in the production and processing stages of wood products, the results showed that the reduction in carbon emissions was approximately twelve times the amount of emissions from the HWP producing and processing stage during the last decade. Furthermore, the net carbon removal and reduction increased from 23 TgC in 1960 to 76.1 TgC in 2014. The annual contribution of HWP could compensate for approximately 2.9% of the national CO_2_ emissions from energy consumption.

Amounts of wood-based materials in international trade of final products are unacquirable in FAO statistics and, in order to avoid double-counting, those that are focused on semi-finished products (sawnwood, wood-based panels and paper) could not be used in the PA method. To increase the contribution of HWP to carbon emission mitigation, we will include the evaluation of carbon substitution efficiency as well as the establishment of a carbon accounting, measurement, and monitoring system in our future research.

## Appendix A

**Table A1:** Conversion factors for the HWP categories and the subcategories.

HWP Categories	Density	Carbon Fraction	Value
Carbon Conversion Factor	**(oven-dry tonne·m^−3^)**	-	**(tonne C·m^−3^)**
Industrial Roudwood (*Aggregate*)	0.52	0.5	0.260
Coniferous	0.45	0.5	0.225
Non-Coniferous	0.59	0.5	0.295
Sawnwood (*Aggregate*)	0.458	0.5	0.229
Coniferous	0.45	0.5	0.225
Non-Coniferous	0.56	0.5	0.280
Wood-Based Panels (*Aggregate*)	0.595	0.454	0.270
Hardboard	0.788	0.425	0.335
Insulating Board	0.159	0.474	0.075
Particle Board	0.596	0.451	0.269
Plywood	0.542	0.493	0.267
Veneer Sheets	0.505	0.5	0.253
Wood Fuel and Residue (*Aggregate*)	0.52	0.5	0.260
Coniferous	0.45	0.5	0.225
Non-Coniferous	0.59	0.5	0.295
Carbon Conversion Factor	**(oven-dry tonne air dry·tonne^−1^)**	-	**(tonne C air dry·tonne^−1^)**
Paper and Paperboard	0.9	0.428	0.385
Wood Charcoal	0.9	0.85	0.765

Source: Factors of industrial roundwood and wood fuel and residues are obtained from 2006 Intergovernmental Panel on Climate Change (IPCC) Guidelines for National Greenhouse Gas Inventories [1]. Factors of sawnwood and wood-based panels are obtained from 2013 Revised Supplementary Methods and Good Practice Guidance Arising from the Kyoto Protocol [15].

**Table A2:** Carbon emission intensity and substitution factor for HWP carbon balance system.

Item	Value
Carbon Emission Intensity	(KgC·m^−3^)
Industrial Roundwood Production	1.58
Sawnwood Processing	2.16
Plywood Processing	12.42
Woodchip Processing	0.31
Carbon Substitution Factor	-
Substitute for Coal	0.96
Substitute for Oil	0.79
Substitute for Natural Gas	0.56

Source: Calculated from technical paper of estimation, reporting and accounting of harvested wood products.

## References

[1] Intergovernmental Panel on Climate Change (IPCC) (2006). Chapter 12: Harvest wood products. 2006 IPCC Guidelines for National Greenhouse Gas Inventories.

[2] Richard S., Martin J., Paul M., Andre F. (2013). The GHG contribution of the cascaded use of harvested wood products in comparison with the use of wood for energy—A case study on available forest resources in Canada. Environ. Sci. Policy.

[3] Winjum J.K., Sandra B., Bernhard S. (1998). Forest harvests and wood products: Sources and sinks of atmospheric carbon dioxide. Forest Sci..

[4] Goodale C.L., Michael J., Apps R.A., Birdsey C.B., Field L.S., Heath R.A., Houghton J.C., Jenkins G.H., Kohlmaier W.K., Liu S.R. (2002). Forest carbon sinks in the Northern Hemisphere. Ecol. Appl..

[5] Hashimoto S., Nose M., Obara T., Moriguchi Y. (2002). Wood products: Potential carbon sequestration and impact on net carbon emissions of industrialized countries. Environ. Sci. Policy.

[6] Pingoud K., Perälä A.L., Pussinen A. (2001). Carbon dynamics in wood products. Mitig. Adapt. Strateg. Glob. Chang..

[7] Skog K.E. (2008). Sequestration of Carbon in harvested wood products for the United States. Forest Prod. J..

[8] Woodbury P.B., James E.S., Linda S.H. (2007). Carbon sequestration in the U.S. forest sector from 1990 to 2010. Forest Ecol. Manag..

[9] Krankina O.N., Harmon M.E., Winjum J.K. (1996). Carbon storage and sequestration in the Russian forest sector. Ambio (Stockholm).

[10] Ji C.Y., Yang H.Q., Nie Y., Hong Y.X. (2013). Carbon sequestration and carbon flow in harvested wood products for China. Int. Forest Rev..

[11] Green C., Avitabile V., Farrell E.P., Byrne K.A. (2006). Reporting harvested wood products in national greenhouse gas inventories: Implications for Ireland. Biomass Bioenergy.

[12] Dias A.C., Louro M., Arroja L., Capela I. (2007). Carbon estimation in harvested wood products using a country-specific method: Portugal as a case study. Environ. Sci. Policy.

[13] Choi S.I., Kang H.M. (2007). The Change in Carbon stocks and emissions assessment of harvested wood products in Korea. J. Korean Forest.

[14] Tonn B., Marland G. (2007). Carbon sequestration in wood products: A method for attribution to multiple parties. Environ. Sci. Policy.

[15] Intergovernmental Panel on Climate Change (2014). 2013 Revised Supplementary Methods and Good Practice Guidance Arising from the Kyoto Protocol.

[16] White M.K., Gower S.T., Ahl D.E. (2005). Life cycle inventories of roundwood production in Northern Wisconsin: Inputs into an industrial forest carbon budget. Forest Ecol. Manag..

[17] Chihiro K., Yuko T., Hideshi N., Mario T. (2014). Carbon balance assessments of harvested wood products in Japan taking account of inter-regional flows. Environ. Sci. Policy.

[18] Zhang X.B., Yang H.Q. (2015). Dynamic projection of storage efficiency and carbon pool structure of China’s harvested wood products based on GFPM. Resour. Sci..

[19] Sathre R., Gustavsson L. (2006). Energy and carbon balances of wood cascade chains. Resour. Conserv. Recy..

[20] Schlamadinger B., Apps M., Bohlin F., Gustavsson L., Jungmeier G., Marland G., Pingoud K., Savolainen I. (1997). Towards a standard methodology for greenhouse gas balances of bioenergy Systems in comparison with fossil energy systems. Biomass Bioenergy.

[21] Yang H., Zhang X. (2016). A rethinking of the production approach in IPCC: Its objectiveness in China. Sustainability.

